# Neoadjuvant Treatment for Pancreatic Adenocarcinoma: A False Promise or an Opportunity to Improve Outcome?

**DOI:** 10.3390/cancers13174396

**Published:** 2021-08-31

**Authors:** Shelize Khakoo, Angelica Petrillo, Massimiliano Salati, Abdul Muhith, Jessica Evangelista, Silvia Seghezzi, Fausto Petrelli, Gianluca Tomasello, Michele Ghidini

**Affiliations:** 1 Department of Medicine, Royal Marsden Hospital, Sutton, Surrey SM2 5PT, UK; Abdul.Muhith@rmh.nhs.uk; 2 Division of Medical Oncology, Department of Precision Medicine, Università degli Studi della Campania Luigi Vanvitelli, 80131 Naples, Italy; angelic.petrillo@gmail.com; 3 Oncology Unit, Ospedale del Mare, 80147 Naples, Italy; 4 Department of Oncology, University Hospital of Modena and Reggio Emilia, 41125 Modena, Italy; massimiliano.salati@unimore.it; 5 Department of Thoracic Surgery, Fondazione Policlinico Universitario A. Gemelli IRCCS, 00168 Rome, Italy; Jessica.evangelista@guest.policlinicogemelli.it; 6 Nuclear Medicine Unit, ASST Bergamo Ovest, 24047 Treviglio, Italy; Silvia_seghezzi@asst-bgovest.it; 7 Oncology Unit, Medical Sciences Department, ASST Bergamo Ovest, 24047 Treviglio, Italy; fausto_petrelli@asst-bgovest.it; 8 Medical Oncology Unit, Fondazione IRCCS Ca’ Granda Ospedale Maggiore Policlinico, 20122 Milan, Italy; gianluca.tomasello@policlinico.mi.it

**Keywords:** pancreatic ductal adenocarcinoma, neo-adjuvant chemotherapy, radiotherapy, immunotherapy, biomarkers, resectability

## Abstract

**Simple Summary:**

Pancreatic cancer usually presents late when it has spread to distant sites. In a small proportion of patients, the cancer can be removed by surgery. Surgery is usually followed by chemotherapy, as studies have shown that this improves survival. However, due to complications after surgery and a decline in fitness, some patients do not start their chemotherapy and many do not complete the planned course. The cancer returns in the majority of patients. Chemotherapy or a combination of chemotherapy and radiotherapy before surgery are being investigated to improve survival. The best treatment regime and patient selection for different treatment strategies remains to be defined and is discussed here.

**Abstract:**

Pancreatic ductal adenocarcinoma (PDAC) has an aggressive tumor biology and is associated with poor survival outcomes. Most patients present with metastatic or locally advanced disease. In the 10–20% of patients with upfront resectable disease, surgery offers the only chance of cure, with the addition of adjuvant chemotherapy representing an established standard of care for improving outcomes. Despite resection followed by adjuvant chemotherapy, at best, 3-year survival reaches 63.4%. Post-operative complications and poor performance mean that around 50% of the patients do not commence adjuvant chemotherapy, and a significant proportion do not complete the intended treatment course. These factors, along with the advantages of early treatment of micrometastatic disease, the ability to downstage tumors, and the increase in R0 resection rates, have increased interest in neo-adjuvant treatment strategies. Here we review biomarkers for early diagnosis of PDAC and patient selection for a neo-adjuvant approach. We also review the current evidence for different chemotherapy regimens in this setting, as well as the role of chemoradiotherapy and immunotherapy, and we discuss ongoing trials.

## 1. Introduction

Pancreatic ductal adenocarcinoma (PDAC) is the 14th commonest cancer worldwide. Amongst all malignancies, PDAC accounted for 2.6% of new cancer cases and 4.7% of new cancer deaths in 2020 [1]. Globally, North America, Europe, and Argentina have the highest incidence of PDAC [2]. PDAC is projected to be the second leading cause of death by 2030 and is currently the fourth most common cause of cancer deaths in Europe [3,4,5].

Tumorigenesis in PDAC is driven by signature gene mutation of *KRAS* in 90% of cases, with frequent inactivation of tumor suppressor genes such as *TP53*, *SMAD4,* and *CDKN2A* and with complex interactions with the tumor microenvironment [6]. PDAC remains a difficult to treat cancer, and the reasons for this are multi-factorial and include late patient presentation due to non-specific symptoms coupled with a propensity for rapid clinical decline, challenges associated with local drug delivery due to a desmoplastic stroma, and aggressive disease biology with a degree of resistance to chemotherapy and radiotherapy, as well as being a tumor with relatively low immunogenicity.

To date, curative resection offers the only chance of cure. However, only 10–20% of patients present with resectable disease, while the majority are diagnosed with borderline resectable, locally advanced (30%), or metastatic disease (50%) [7]. In order to assess resectability, a triple-phase staging contrast-enhanced computed tomography (CT) scan of the abdomen and pelvis should be performed for all patients to assess the anatomic link between the primary tumor and vascular structures and to assess for the presence of intra-abdominal metastases. There are variations in the definition of resectability, but key anatomical criteria such as involvement of the superior mesenteric artery and superior mesenteric vein/portal vein invasion can help differentiate resectable, borderline resectable, and locally advanced PDAC [8,9]. The National Comprehensive Cancer Network (NCCN) guidelines are commonly used to define resectability and are summarized in Table 1 [10]. Since their initial publication, the International Association of Pancreatology international consensus definition was derived for borderline resectable disease and includes biological and conditional criteria in addition to anatomical criteria [11]. They noted that a carbohydrate antigen (CA)-19.9 > 500 units/mL and regional lymph node metastasis on PET-CT or biopsy-proven lymph node metastasis were associated with a worse prognosis and likely represented disseminated metastatic disease, even in the absence of clear radiological findings. Therefore, they recommend that in the presence of these features, a tumor that is resectable by anatomical criteria should be deemed borderline resectable. Other commonly utilized resectability classifications include those from the American Hepato-Pancreato-Biliary Association (AHPBA)/Society of Surgical Oncology (SSO)/Society for Surgery of the Alimentary Tract (SSAT) [12], the University of Texas MD Anderson Cancer Center (MDACC) [13,14], and the Alliance for Clinical Trials in Oncology group [15].

Despite advances in surgical techniques, along with the incorporation of more active chemotherapy regimens, the 5-year survival rate for PDAC has remained poor at 9% for all disease stages combined [16]. After curative intent surgery, the National Cancer Database reported that 5-year OS was only 23.4% [17]. Given that PDAC has a high propensity to metastasize, thereby suggesting the presence of micrometastatic disease, studies began to investigate the role of adjuvant chemotherapy, which is now an established standard of care (Table 2). Chemotherapy regimens that were shown to be active in the metastatic setting were investigated in the adjuvant setting. In fit patients, modified FOLFIRINOX currently provides the option with the best survival data, with a 3-year OS rate of 63.4% in the PRODIGE-24 study [18]. Interestingly, although the combination of gemcitabine and nanoparticle albumin-bound (nab) paclitaxel represents a first-line treatment option in the metastatic setting based on the results of the MPACT study [19], the adjuvant APACT study, which investigated this combination chemotherapy, did not meet its primary endpoint of disease-free survival (DFS), although OS was improved over gemcitabine monotherapy [20,21].

The benefit from adjuvant chemotherapy in patients undergoing a curative treatment paradigm is undisputed. However, 50% of patients may never commence adjuvant chemotherapy due to post-operative complications or poor performance status, and a proportion of patients do not complete the intended course [30,31]. Additionally, with increasing time from surgery to chemotherapy initiation, there may be a reduction in the survival benefit [32], and this is of particular relevance in patients who do not complete the full planned course of adjuvant chemotherapy [33]. These factors, along with other theoretical advantages such as earlier treatment of micro-metastatic disease, the potential to downstage tumors with improved R0 resection rates, and the opportunity to study disease biology prior to subjecting patients to major surgery, led to increased interest in neo-adjuvant treatment strategies. However, the requirement for a histological diagnosis prior to commencing chemotherapy and the possibility of treatment toxicities precluding surgery are potential disadvantages of a neo-adjuvant treatment approach that should be taken into consideration. Whilst delaying surgery could also be seen as a potential disadvantage due to the window of opportunity that has been lost in cases of disease progression, the likelihood is that in such cases, patients have aggressive disease biology or more advanced disease than initially thought and have been spared from extensive surgery and its associated risks. Therefore, this should not be regarded as a drawback per se. Data from the ESPAC-4 adjuvant study demonstrated that risk factors for local relapse included post-operative CA-19.9 (hazard ratio (HR) 1.32) and N2 status (HR 2.16), while risk factors for distant relapse included N1 status (HR 1.76) and N2 status (HR 2.81). Risk factors for survival included R status (HR 1.27), maximum tumor size (HR 1.11), post-operative CA-19.9 (HR 1.32), N1 (HR 1.44), and N2 status (HR 2.1) [34]. These data provide further supportive evidence for a neo-adjuvant treatment strategy in order to downstage tumors to improve outcome. Given the importance of margin status on prognosis, surgery should ideally be performed in high volume centers with expertise.

Although less well studied than the adjuvant setting, emerging evidence suggests that select populations may be more likely to benefit from neo-adjuvant treatment, thereby suggesting that treatment will need to be tailored for individual patients. Additionally, given that the success of combination treatment with gemcitabine and nab-paclitaxel in the metastatic setting did not directly translate into the adjuvant setting, the question of which chemotherapy regimen is best in the neo-adjuvant setting needs to be carefully considered.

Here we review the evidence for different neoadjuvant treatment regimens, including chemotherapy, chemo-radiotherapy, and immunotherapy, as well as biomarkers for early diagnosis, and we discuss which patients may benefit from neo-adjuvant treatment.

## 2. Biomarkers

### 2.1. Diagnostic Biomarkers

There is a clear unmet need to discover biomarkers to aid the early diagnosis of PDAC, given that patients usually become symptomatic and present later in the clinical course when curative resection is no longer feasible. Additionally, any biomarker used in this context, needs to be able to reliably differentiate PDAC from both chronic pancreatitis and other benign or malignant diseases [35]. Currently, diagnosis using computed tomography (CT) or magnetic resonance imaging (MRI) is not completely specific, while a cytological or histological diagnosis is often hard to obtain, and the blood markers carcino-embryonic antigen (CEA) and CA-19.9 harbor poor specificity (68–80%) and sensitivity (80%) [36].

Multiple studies have evaluated the performance of CA-19.9 with other protein biomarkers. CA-19.9 was evaluated together with apolipoprotein A-IV and metalloproteinase-1 in a cohort of 182 individuals including stage I–II and III–IV PDAC patients, patients with pancreatitis, and healthy controls. Sensitivity reached a value of 86%, specificity was 90%, while sensitivity was 71% for CA-19.9 alone [37]. Moreover, elevated levels of plasma thrombospondin-2 combined with CA-19.9 were able to discriminate between PDAC patients and healthy controls, with a sensitivity of 87% and a specificity of 98% [38]. Another panel included apolipoprotein E, apolipoprotein A-I, apolipoprotein L1, and inter-alpha-trypsine inhibitor heavy chain H3 in combination with CA19-9. The cohort included 80 PDAC patients, 30 with benign pancreatic disease, and 40 healthy controls. Sensitivity (95%) and specificity (94.1%) were significantly increased with respect to CA-19.9 alone [39]. A glycan called sTRA (sialylated tumor-related antigen) was shown to be produced and secreted by pancreatic tumors and patient-derived xenograft that did not express CA-19.9. Two biomarker panels were tested, one including CA19-9 and two versions of the sTRA assays and another with two sTRA assays. Both panels significantly improved performance over CA-19.9 alone in PDAC diagnosis (*p* < 0.001) [40].

With improvements in precision medicine, the use of blood and other body fluids as liquid biopsy tools for diagnosis, genotyping, prognostication, response assessment, and surveillance has gained increasing interest in oncology. In this regard, circulating tumor DNA (ctDNA) can act as a genomic surrogate for tumor and has been investigated as an early diagnostic biomarker for PDAC, given that >90% of tumors harbor a *KRAS* mutation. In early stage PDAC, ctDNA was only detected in 30–65% of patients [41,42,43,44,45], while detectability improved to 70–80% in cases of more advanced disease [45,46]. However, it should also be noted that when using *KRAS* mutation, ctDNA has also been detected in up to 20% of cases with chronic pancreatitis and 17% of non-malignant pancreatic masses [47]. Therefore, this marker cannot reliably be used for early diagnosis, although detection of ctDNA does appear to be associated with worse prognosis. The role of ctDNA in the neo-adjuvant setting may be best utilized as a non-invasive method of real-time monitoring of response to chemotherapy, given that scans cannot be performed too often due to radiation exposure. This would only be a viable option in patients who have upfront detectable ctDNA, but it may help tailor neo-adjuvant treatment strategies for patients. In patients with a high baseline ctDNA value, this may raise suspicion of more widely disseminated disease than is perhaps visible on imaging. Although ctDNA is a rapidly evolving biomarker, further studies are needed to confirm clinical utility and its precise role in PDAC.

Exosomes, which contain proteins and enzymes, constitute a class of extracellular vehicles defined as membrane-bound nanovesicles of endocytic origin, with a diameter of 40–150 nm. Increasing evidence suggests that exosomes are associated with cancer and accelerate PDAC cell proliferation, migration, and invasion. Unlike ctDNA, exosomes do not depend on the occurrence of apoptosis or necrosis to be detectable and have a much longer half-life [46]. Moreover, their contents maintain a signature reflective of their cell of origin, which makes them ideal candidates for early diagnosis. A meta-analysis showed that searching for exosomes is more useful for early detection of PDAC than ctDNA and circulating cells [48]. They can intervene in the sequestration of cytotoxic drugs, thereby reducing the effective drug concentration at target sites. In addition, they may capture monoclonal antibodies intended to target receptors at the cell surface. Moreover, resistant tumor cells can deliver mRNA, miRNA, long noncoding RNA, and protein, inducing resistance in sensitive cells. They also mediate cross-talk between cancer cells and stromal cells in the tumor microenvironment, leading to tumor progression and acquisition of therapeutic resistance. Extracellular vesicles are also promising tools for predicting chemoresistance in cancers [49].

Some microRNAs (miRNAs) were reported to be over-expressed in the plasma of patients with early-stage PDAC compared with normal controls and may therefore help with early diagnosis. For example, miR-16 showed a 92% sensitivity and 95.6% specificity for the determination of early PDAC cases. In addition to miR-16, compared with controls, miR-155, miR-181a, miR-181b and miR-210 were also found to be overexpressed in PDAC patients. MiR-19a-3p was shown to be overexpressed in PDAC with a potential to be used as an early non-invasive diagnostic biomarker but also as a prognostic biomarker. Another study reported the role of miR-29a, miR29b, miR-103, and miR-320 as early diagnostic predictors of PDAC when upregulated [50].

### 2.2. Prognostic Biomarkers and Predictors of Resectability

In patients with resectable PDAC, high pre-operative C-reactive protein-to-albumin ratio was reported as an independent predictor of poor survival after resection of the primary tumor [51]. Similarly, two single-nucleotide polymorphisms of genes regulating cancer progression, invasion, and metastasis (*CHI3L2* and *CD44*) were associated with poor survival after PDAC resection. Prognostic biomarkers may be useful in selecting patients at high-risk of relapse after surgery for a neo-adjuvant chemotherapy strategy in an attempt to treat micro-metastatic disease early [52]. However, any benefit from such an approach remains unproven, and it could be argued that such patients have an aggressive disease biology and may not be suitable for a curative treatment paradigm if these biomarkers are merely an early reflection of disseminated disease.

In borderline-resectable patients receiving neo-adjuvant chemotherapy, the expression of marginal zone B and B1-cell-specific protein (MZB1) was detected in the tumor stroma. MZB1 was associated with higher accumulation of CD8+ tumor-infiltrating lymphocytes induced by chemotherapy. Patients with higher accumulation of CD8+ cells after chemotherapy had better prognosis after neo-adjuvant treatment and resection [53]. Similarly, sTRA was evaluated both in tumor tissue and the plasma of patients receiving neo-adjuvant chemotherapy. High levels of sTRA were associated with absence of benefit from neo-adjuvant treatment and rapid relapse following pre-operative chemotherapy. Thus, the authors proposed that in patients with localized PDAC, sTRA could be used as a marker to differentiate patients who should avoid curative surgery due to the increased likelihood of rapid relapse [54].

So far, none of these candidate biomarkers have been approved for clinical practice. Indeed, results obtained in small investigational cohorts need to be validated in larger studies. However, this is often challenging due to the long follow-up times required and the need to recruit a large number of patients for conclusive results. Additionally, PDAC is a heterogeneous disease with different tumor clones that may contribute to inter-patient variability in the expression of tissue and circulating biomarkers [35].

It has been suggested that the simple dichotomy of resectable vs. borderline resectable disease can be overcome by biological classification of favorable or unfavorable disease according to biomarker status. Indeed, a recent study that conducted multi-omics profiling of resectable PDAC revealed molecular subtypes with distinct tumor biology [55]. Therefore, even within the resectable category alone, there are likely to be PDACs that are destined to behave more aggressively. The classification of basal-like and classical PDAC, with basal-like behaving more aggressively, appears to be widely accepted, although there is still significant heterogeneity within these groups. This has led to a further breakdown to basal-like A, basal-like B, classical-like A, and classical-like B [56]. Overall, the classical groups appear to be enriched in resectable disease. The basal-like A subgroup, which is rare in resectable disease, responds the least to gemcitabine-based chemotherapy or mFOLFIRINOX. Based on these data, subtyping may be a way to help differentiate which patients should be treated with neo-adjuvant chemotherapy.

Some centers consider nearly all patients who appear to have resectable PDAC as having borderline resectable disease, given the inaccuracy of imaging and the very high rates of a positive margin after surgery and its association with poor OS. Older trials and meta-analysis included patients staged with substandard imaging modalities or sub-optimal chemotherapy schedules (e.g., gemcitabine alone or platinum analogues). Modern chemotherapy (e.g., FOLFIRINOX) allows significant shrinkage and conversion surgery in about 60% of patients with borderline resectable PDAC [57,58], so it is more widely adopted compared with years ago. Currently, there is a lack of well-defined, reliable, biological and/or anatomical biomarkers to predict curative resection or the risk of micro-metastatic disease. In a large cohort of resected PDACs that underwent adjuvant chemotherapy, lymph-node status and pre-operative CA-19.9 status were independent predictors of 5-year survival, confirming that anatomical (locoregional staging) and humoral markers (CA-19.9) are crucial for defining prognosis and identifying a potentially cure [59]. In another large study of resected PDAC, pre-operative CA-19.9 levels correlated with resectability and 5-year survival. Notably, 5-year survival was 0% when baseline CA-19.9 was >1000 U/mL [60]. Generally, despite low specificity, CA-19.9 baseline values are usually predictors of outcome. Additionally, among patients who appear to have potentially resectable PDAC, pre-operative CA-19.9 level can help to predict the presence of radiographically occult metastatic disease, the chance of an R0 resection, and long-term survival [61].

Radiological modalities, surgical exploration, metabolic and functional imaging, and plasma biomarkers have been explored as predictors of resectability. Disease is usually split between resectable and borderline resectable/unresectable cases. With conventional imaging (CT), the distinction between these two categories is associated with a high sensitivity but reduced specificity (high false-positive rates) [62]. Staging laparoscopy is another way to confirm the operability of a PDAC defined as resectable on imaging. In a systematic review and meta-analysis, including 12 studies reporting outcomes on 1756 patients with resectable disease after standard imaging, 20% of cases were deemed unresectable by staging laparoscopy [63].

Functional metabolic imaging such as FDG-PET has been shown to be a useful measure of radiological response as well as for initial staging. In a subset of 194 undergoing total neo-adjuvant therapy for borderline resectable or locally advanced PDAC, three factors were independently associated with survival: extended chemotherapy duration (≥6 cycles), optimal post-chemotherapy CA-19.9 response, and a major pathological response. Major pathologic response was defined as complete pathological response or near complete response according to the College of American Pathology criteria. In a subset of patients with interval metabolic (PET) imaging after initial chemotherapy, complete metabolic response highly correlated with major pathological response [64]. In a systematic review of imaging modalities able to predict response and resectability, two studies focused on PET response [65]. They showed a significant correlation between the reduction in SUVmax and resectability.

The UK National Institute for Health and Care Excellence (NICE) has recommended the use of PET-CT in the routine staging of patients with resectable PDAC [66]. Despite the limitations of FDG-PET in PDAC (intrinsic uptake, operator-dependent SUV measurement), in a prospective study assessing the diagnostic accuracy and clinical value of PET-CT in suspected PDAC patients, Ghaneh et al. showed that in cases initially identified as having resectable tumors on CT, PET-CT changed management in 45% of patients and prevented unnecessary resections in 21% of patients scheduled for surgery [67].

Finally, endoscopic ultrasound (EUS) is another valuable modality for the staging of PDAC. Multiple studies comparing EUS with other imaging modalities for initial diagnosis and staging of pancreatic cancer concluded that EUS might be more accurate for smaller tumors, local T and N staging, and predicting vascular invasion. The development of modern CT has markedly improved sensitivity for the detection of smaller tumors and the presence of vascular invasion, apart from dictating resectability [68]. However, EUS-guided fine-needle aspiration biopsy remains the best method for obtaining a tissue diagnosis.

In conclusion, in the presence of a pancreatic mass suspected as being a PDAC, a CT scan and EUS (for tissue confirmation) are appropriate steps to assess disease extent and resectability. Biomarkers and clinical status (CA-19.9, ECOG PS) may assist in prognostication. With pre-operative therapy, imaging may not reliably show whether viable tumor continues to persist (fibrosis); however, imaging can help identify disease progression, including the development of distant metastases, which would preclude surgery. It is also important to consider patient-related factors such as performance status and change in CA-19.9 levels as markers of response to neo-adjuvant therapy and likely benefit from attempted surgical resection. Minimal radiological progression or a stable disease picture may not per se represent a criterion to refuse surgical exploration. In some cases of PDAC where the resectability status is less clear, the addition of PET and/or laparoscopy may help determine whether neo-adjuvant treatment is preferable over upfront surgery.

## 3. Neo-Adjuvant Treatment Strategies

### 3.1. The Role of Neo-Adjuvant Chemoradiotherapy

The addition of radiotherapy to chemotherapy has been explored in the neo-adjuvant setting in an attempt to improve R0 resection rates and ultimately survival outcomes in both resectable and borderline resectable disease, and key studies are summarized in Table 3. Among the advantages of pre-operative chemoradiotherapy are the likelihood of better tolerance, the chance of achieving tumor downsizing and downstaging, the availability of well-defined tumor volume, and better-oxygenated tissues. However, the increase in post-surgical complications and the chance of early tumor progression thereby limiting the potential for surgery are potential drawbacks of neo-adjuvant chemoradiotherapy that need to be taken into account.

In recent years, evidence has become increasingly available suggesting a survival benefit for neo-adjuvant chemo(radio)therapy in resectable PDAC and borderline resectable PDAC. A large population-based study from the Surveillance, Epidemiology, and End Results (SEER) database, including nearly 4000 patients, showed a statistically significant prolongation in OS for resectable PDAC patients receiving neo-adjuvant radiotherapy (23 months) compared with those receiving adjuvant radiotherapy (17 months) or no radiotherapy (12 months) [69]. More recently, in a retrospective single-institution propensity score-adjusted analysis from the MD Anderson Cancer Center, neo-adjuvant radiotherapy produced higher R0 resection rates and lower rates of lymph node positivity and local recurrences compared with pre-operative chemotherapy alone in 258 patients with resectable PDAC [70]. Contrary to these findings, in a meta-analysis that demonstrated an improvement in survival for pre-operative treatment compared with immediate surgery (18.8 vs. 14.8 months), an inferior survival was reported when radiotherapy was added to chemotherapy compared with chemotherapy alone (17.8 vs. 20.0 months) in a subset analysis [71]. Notably, heterogeneity of the included studies with regard to radiation doses and schedules and chemotherapy regimens precludes any definitive conclusions from being drawn. Historically, most of the studies evaluating the role of radiotherapy pre-operatively were retrospective and/or non-randomized, enrolling mainly heterogeneous, small patient populations. Furthermore, these trials suffered from inherent bias related to the reporting of survival data for patients who underwent pancreatic resection, while excluding those not amenable for surgery from the analyses. This makes the interpretation and comparison of the studies challenging and the available evidence weak and of low quality.

The long-term results of the PREOPANC-1 trial, which is the first multicenter, randomized phase III trial investigating pre-operative chemoradiotherapy in PDAC, have been reported at the American Society of Clinical Oncology (ASCO) 2021 Meeting [72,73]. In this study, 246 patients with resectable PDAC or borderline resectable PDAC were randomly allocated to upfront surgery and adjuvant gemcitabine or pre-operative gemcitabine-based chemoradiotherapy (36 Gy in 15 fractions) and surgery followed by adjuvant gemcitabine. The chemoradiotherapy group had 1 cycle of gemcitabine chemotherapy before and 1 cycle after the chemoradiotherapy. With a median follow-up of 59 months, a significant improvement in survival was shown favoring chemoradiotherapy, with a median OS and 5-year OS of (15.7 vs. 14.3 months) and (20.5% vs. 6.5%), respectively. Among resected cases, the benefit for neo-adjuvant chemoradiotherapy was even greater, with a median OS and 5-year OS of (33.7 vs. 17.3 months) and (33.9% vs. 8.4%), respectively. The secondary study end-points were met as DFS (HR, 0.70; *p* = 0.009), locoregional failure-free interval (HR, 0.57; *p* = 0.004), and R0 resections (72% vs. 43%, *p* < 0.001) were all superior with pre-operative chemoradiotherapy. Regarding the safety profile, the proportion of patients experiencing G3–G4 adverse events was similar between treatment arms (*p* = 0.096). Moreover, the compliance of intended pre-operative chemoradiotherapy (ITT, 76% vs. 51%) was better than that of intended post-operative chemotherapy in the immediate surgery group (ITT, 51%), and pre-operative chemoradiotherapy had a higher chance of being completed (89% vs. 58%). Borderline resectable PDAC seems to benefit more from pre-operative chemoradiotherapy, based on a predefined subgroup analysis, although an interaction test of hazard rates between both groups was not significant. While the results of this study clearly support a neo-adjuvant treatment strategy, unanswered questions remain. It is still unclear whether the optimal chemoradiotherapy strategy is gemcitabine-based treatment. Additionally, the amount that the chemoradiotherapy contributed to the favorable outcome is unclear, as these patients also received 2 cycles of chemotherapy with gemcitabine neo-adjuvantly.

Based on the effectiveness displayed in the advanced-disease setting, the mFOLFIRINOX regimen has been rapidly introduced in earlier disease stages with or without pre-operative radiotherapy. To this end, mFOLFIRINOX followed by (chemo)radiotherapy as a total neo-adjuvant approach has been shown to be feasible in borderline resectable PDAC, resulting in promising oncological outcomes. In a single-arm phase II trial, after 8 cycles of neo-adjuvant mFOLFIRINOX, patients received either short- or long-course radiotherapy depending on vascular involvement upon restaging, with high R0 resection rates (97%, among patients undergoing surgery), and median progression-free survival (mPFS) was 48.6 months (95% CI, 14.4 to not reached), while median OS has not yet been reached [74]. However, the results of the phase II randomized Alliance A021501 trial have recently been presented at the 2021 ASCO Gastrointestinal Cancer Symposium [75] and were disappointing for the addition of radiotherapy into the neo-adjuvant treatment paradigm. In this study, 126 patients with borderline resectable PDAC were randomly assigned to receive either pre-operative mFOLFIRINOX for 8 cycles (arm A) or pre-operative mFOLFIRINOX for 7 cycles, followed by 5 days of hypofractionated radiotherapy (arm B) using either stereotactic body radiotherapy (SBRT, 33–40 Gy in 5 fractions) or image-guided radiotherapy (25 Gy in 5 fractions). In both groups, patients without disease progression underwent pancreatectomy followed by 4 cycles of adjuvant chemotherapy with mFOLFOX6. The primary endpoint was the 18-month OS rate of each arm evaluated independently relative to historical data. The addition of hypofractionated radiotherapy after neo-adjuvant mFOLFIRINOX failed to exceed the 50% OS threshold at 18 months (47.3%), and the radiotherapy-containing arm had to be closed early due to the low number of patients who proceeded to pancreatectomy based on an interim futility analysis. Contrary to this, the chemotherapy-alone arm completed accrual and showed an overall 18-month OS of 66.4%, and among the 49% of patients who proceeded to pancreatectomy following neo-adjuvant therapy, the R0 resection rate was 88%, and the 18-month OS rate reached 93.1%. Whilst the radiotherapy-containing arm produced disappointing results, the role of radiotherapy in the neo-adjuvant setting should not yet be dismissed. The single-arm phase II trial [74] demonstrated some encouraging results, and the fact that the results do not concur with the findings of the Alliance A021501 study likely reflects the heterogeneous nature of PDAC and the need for us to better identify which patients benefit from the various different treatment strategies under investigation.

The role of pre-operative (chemo) radiation in both resectable PDAC and borderline resectable PDAC is still strongly debated, and the highest level of evidence still favors surgical resection followed by adjuvant chemotherapy in this setting. The PREOPANC-1 study forms an important contribution to the evidence base in support of neo-adjuvant chemoradiotherapy, as it represents the first large phase III randomized trial in this space. Nonetheless, more data are needed, and future and ongoing trials incorporating radiotherapy in neo-adjuvant treatment strategies will hopefully aid in better clarifying its role in resectable PDAC and borderline resectable PDAC. Ongoing studies will hopefully address questions regarding the role of radiotherapy (non-SBRT) and the optimal systemic treatment to be given. These issues are under active investigation in the ongoing PREOPANC-2 (EudraCT: 2017-002036-17) and PRODIGE 44 trials (ClinicalTrials.gov Identifier: NCT02676349). The former is comparing neo-adjuvant gemcitabine-based chemoradiotherapy and adjuvant gemcitabine vs. total neo-adjuvant mFOLFIRINOX in borderline resectable PDAC and resectable PDAC, while the latter is evaluating neo-adjuvant mFOLFIRINOX followed by the addition or omission of chemoradiotherapy and adjuvant chemotherapy after surgery in patients with borderline resectable PDAC.

### 3.2. Neo-Adjuvant and Perioperative Chemotherapy

The drive for neo-adjuvant and peri-operative treatment in PDAC is based on the fact that this tumor has a propensity to metastasize early, thereby suggesting the presence of micrometastatic disease that requires early intervention. PDACs are often diagnosed as locally advanced or borderline resectable tumors; in those cases, an induction treatment is recommended according to international guidelines in order to increase the rate of optimal resections [7].

FOLFIRINOX and gemcitabine/nab-paclitaxel represent the most active chemotherapy regimens investigated in PDAC in the metastatic setting over the last decade [19,77]. In the neo-adjuvant setting, a meta-analysis of 24 trials (6 retrospective, 8 prospective phase I–II) evaluated the role of FOLFIRINOX in 1802 borderline resectable PDACs [58]. The analysis demonstrated a resection rate of 67.8% (95% confidence interval (CI): 60.1–74.6%) and an R0 resection rate of 83.9% (95% CI: 76.8–89.1%). The median OS was 11.0–34.2 months; the patient-level median OS was 22.2 months (95% CI = 18.8–25.6 months), and patient-level median PFS was 18.0 months (95% CI = 14.5–21.5 months). The most significant grade 3–4 toxicities were neutropenia (17.5%), diarrhea (11.1%), and fatigue (10.8%). These data confirm the findings from a previously published meta-analysis [78]. Additionally, they represent the strongest evidence regarding the use of FOLFIRINOX in the neo-adjuvant setting since no phase III trials have been specifically published in this setting to date. However, further prospective trials are needed in order to confirm these findings.

Key neo-adjuvant studies in locally advanced PDAC are summarized in Table 4. Nab-paclitaxel and gemcitabine combination chemotherapy was investigated in the phase II GAP trial [79]. The trial randomized 124 locally advanced PDAC patients to receive 3 cycles of gemcitabine with or without nab-paclitaxel as neo-adjuvant treatment. The trial showed a reduction in distant disease progression at 3 and 6 months (3 months: 25.4% in the experimental arm vs. 45.6% with gemcitabine (control arm), *p* = 0.01; 6 months: 20.8% vs. 35.6%). The response rates were 27% and 5.3%, respectively. Median OS was 12.7 and 10.6 months in the experimental and control arm, respectively; median PFS was 7 months in the nab-paclitaxel-containing group compared with 4 months for the gemcitabine group. These encouraging data were subsequently confirmed in the phase II LAPACT trial (open-label, single arm, multicenter), which evaluated the safety and efficacy of six cycles of gemcitabine/nab-paclitaxel combination treatment in 107 locally advanced PDAC patients [80]. The trial showed the following outcomes: median time to treatment failure (primary endpoint) 9.0 months, median progression-free survival 10.9 months, median OS 18.8 months, disease control rate 77.6%, response rate 33.6%. The toxicities were in line with those reported in the GAP trial, and no adjuvant treatment after resection was planned [79].

To date, there are no prospective, validated phase III trial data comparing FOLFIRINOX and the combination of nab-paclitaxel and gemcitabine. However, recently a European multicenter study compared these two regimens in a retrospective analysis [81]. The study included 147 patients with locally advanced unresectable PDAC treated at seven European centers over a period of 8 years (2010–2018). The analysis showed similar outcomes for FOLFIRINOX and gemcitabine/nab-paclitaxel. In particular, tumor resection rates (16.7% vs. 16.1% in nab-paclitaxel and gemcitabine and FOLFIRINOX group, respectively; *p* = 1) and R0 resection rates (88.9%) were similar between the two groups. Additionally, there was no significant difference between either mPFS (9 vs. 12.1 months, respectively; 95% CI: 10.1–14.6; *p* = 0.8) or mOS (15.7 vs. 16.7 months, respectively; 95% CI: 14.8–20.4; *p* = 0.7). These results are in line with those previously reported in the LAPACT trial [80]. Abdominal pain at baseline (HR = 2.03, *p* = 0.03), tumors located in the pancreatic tail (HR = 4.35, *p* = 0.01), CA-19.9 > 200 UI/L (HR = 2.03, *p* = 0.004), and tumor resection (HR = 0.37, *p* = 0.007) were independent prognostic factors for both PFS and OS, whereas CA-19.9 ≤ 200 UI/L (OR = 2.6, *p* = 0.047) was predictive of tumor response. However, the results should be interpreted with caution due to the intrinsic limitations associated with the retrospective nature of the analysis (selection bias, imbalance of patient characteristics, and treatment according to the local drug approval). Additionally, the similar rates of high-grade toxicity reported between the two regimens is not in keeping with previous reports. This could be related to differences in the management of toxicity or different measures used to prevent toxicity (e.g., use of G-CSF for the prevention of febrile neutropenia in the FOLFIRINOX group).

**Figure 1:**
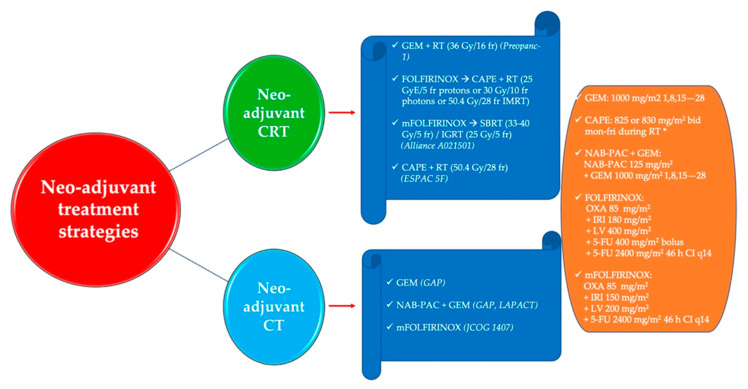
Regimens for neoadjuvant chemoradiotherapy or chemotherapy in patients with pancreatic adenocarcinoma. Legend. CAPE: capecitabine; CI: continuous infusion; CRT: chemoradiotherapy; CT: chemotherapy: GEM: gemcitabine; h: hours; IGRT: image-guided radiotherapy; IMRT: intensity-modulated radiotherapy; IRI: irinotecan; LV: leucovorin; NAB-PAC: nab-paclitaxel; OXA: oxaliplatin; RT: radiotherapy; SBRT: stereotactic body radiation therapy; 5-FU: 5-fluorouracil. * 825 mg/m^2^ in FOLFIRINOX → CAPE + RT trial, 830 mg/m^2^ in ESPAC 5F trial.

The preliminary results of the phase II randomized JCOG1407 trial were recently presented at the ASCO 2021 meeting [82]. The trial randomized 126 Japanese patients to receive modified FOLFIRINOX or gemcitabine/nab-paclitaxel as neo-adjuvant treatment for locally advanced PDAC patients. The trial showed that neo-adjuvant treatment is feasible; however, the data regarding the outcomes are still immature, and therefore the optimal chemotherapeutic strategy in this setting cannot yet be determined (1-year OS rate was in favor of gemcitabine/nab-paclitaxel (82.5% vs. 77.4%), whereas the 2-year OS rate was in favor of modified FOLFIRINOX (48.2% vs. 39.7%)). Nevertheless, this trial represents unique, prospective evidence in this field, and the mature survival data are eagerly awaited. There may be a role for biomarker-driven selection of patients likely to be platinum sensitive and therefore more likely to benefit from modified FOLFIRINOX. Important ongoing studies in locally advanced PDAC include SCALOP-2 (NCT02024009), CONKO-007 (NCT01827553), and DIRECT (NCT03899636), with the latter evaluating the role of nanoknife in this population. Figure 1 shows the main regimens for neoadjuvant chemoradiotherapy or chemotherapy.

Peri-operative chemotherapy for PDAC has mainly been assessed in patients with resectable disease at diagnosis. In this context, the phase II PACT-15 trial randomized patients with resectable PDAC to receive surgery followed by 6 cycles of adjuvant chemotherapy with gemcitabine—arm A; PEXG (cisplatin, epirubicin, gemcitabine, and capecitabine)—arm B; or peri-operative chemotherapy (3 pre- and 3 post-operative cycles of PEXG schedule)—arm C [83]. The trial enrolled 88 patients, showing better 1-year event-free rate in the peri-operative arm (23%, 50%, and 66% in arm A, B, and C, respectively). The R0 resection rate and mOS was also highest in the peri-operative treatment arm. The investigators decided not to conduct the phase III part of the study because the standard of care for adjuvant treatment in resectable PDAC has changed over the years.

The phase II randomized SWOG S1505 trial compared modified FOLFIRINOX and gemcitabine/nab-paclitaxel (12 weeks pre- and 12 weeks post-operative treatment) in 103 patients with resectable PDAC at diagnosis. The preliminary results showed no difference in 2-year OS and resection rate between the two arms (2-year OS: 22.4 vs. 23.6 months; ORR: 77% vs. 73%) [84,85]. However, the final results are awaited.

One of the most innovative strategies in the field of peri-operative approaches is the integration of biomarker research to select patients who can benefit more from this therapeutic paradigm. Based on the results of the COMPASS trial in advanced PDAC [86], the phase II NeoPancONE trial is currently investigating the role of GATA-6 as a potential predictive biomarker in resectable, locally advanced, and metastatic PDAC [87].

In PDAC, 6–7% of patients have a germline mutation in the *BRCA* genes, with a higher incidence in high-risk populations and those with familial syndromes [88]. Patients with a germline mutation in *BRCA* or other DNA repair genes such as *PALB2* are thought to have tumors with increased sensitivity to platinum agents. Increased platinum sensitivity arises because these genes are involved in the homologous recombination repair pathway. In view of this, there is a rationale to ensure that in this patient population, platinum compounds are introduced early in the treatment paradigm. Indeed, in a study evaluating the complete pathological response rate in borderline resectable PDAC, in patients treated with neo-adjuvant FOLFIRINOX, the rate was higher for germline *BRCA* mutation carriers (44.0%) compared with 10% in those who did not have a *BRCA* mutation (*p* = 0.009) [89]. Additionally, DFS was significantly longer in the germline *BRCA* carriers (not reached vs. 7 months, *p* = 0.03), although this did not translate into a statistically significant difference in terms of OS benefit.

Among ongoing trials, the phase II NEO-Nal-IRI trial, which is currently testing the activity of NALIRIFOX (FOLFIRINOX with liposomal irinotecan in place of the standard irinotecan) (NCT03483038), is worthy of being mentioned [90]. The population being recruited includes borderline resectable PDAC and resectable PDAC. The results will be particularly interesting, as liposomal irinotecan is active in the metastatic setting [91].

In conclusion, evidence suggests that both FOLFIRINOX and gemcitabine/nab-paclitaxel chemotherapy are active and may have a role in the pre-operative treatment of borderline resectable and locally advanced tumors. However, the exact role of neo-adjuvant and peri-operative treatment is still not clearly defined for PDAC. This is in part due to trials including heterogeneous populations as well as the lack of consensus regarding the resectability criteria used. Additionally, other limitations include the lack of selection according to molecular classification of PDAC [92], the small number of patients involved in each analysis, and the trial designs, which are often retrospective rather than randomized controlled trials. Therefore, further dedicated prospective randomized trials are needed in order to evaluate the role of neo-adjuvant or perioperative strategies in localized PDAC.

### 3.3. Immunotherapy in the Neo-Adjuvant Setting for PDAC

PDAC is considered to be less immunogenic compared with other neoplasms. Moreover, it is characterized by the presence of the stroma, which represents an active part in the modulation of the complex interactions between tumor and the host immune system, leading to an immunosuppressive tumor microenvironment. Additionally, the proportion of patients with high microsatellite instability (MSI) tumors is very low (1%); these cases are mainly related to the Lynch syndrome. Nevertheless, several preclinical and clinical trials have been developed in order to find some application of immunotherapeutic agents in PDAC.

In the neo-adjuvant setting, there is a limited evidence base for immunotherapy. A recently published phase III trial evaluated the efficacy and safety of algenpantucel-L—a cancer vaccine based on irradiated allogenic transfected PDAC cells—with or without chemotherapy (FOLFIRINOX or gemcitabine/nab-paclitaxel), followed by chemoradiotherapy in 303 patients with borderline resectable or locally advanced PDAC. The mechanism of action was based on the epitope spreading, which amplified the host immune response, leading to the death of PDAC cells by activation of antibody-dependent cell-mediated cytotoxicity. Unfortunately, the trial did not show any benefit in survival by adding algenpantucel-L to the chemotherapy if compared with the control arm (median overall survival 14.3 vs. 14.9 months, respectively; HR: 1.02, *p* = 0.98) [93].

Among vaccines tested in PDAC, GVAX is the most studied. However, all published trials with this agent were conducted in the adjuvant or in the metastatic setting, and no evidence exists in the neo-adjuvant treatment for PDAC patients to date.

In an attempt to improve the immunotherapeutic response in PDAC, immunotherapy alongside chemotherapy has been investigated. The preliminary data regarding the safety and efficacy of the combination of KN046 (a novel recombinant humanized bispecific antibody that blocks PD-1/PDL1 and CTLA-4) and nab-paclitaxel/gemcitabine as first-line treatment for unresectable locally advanced or metastatic PDAC were recently presented at the ASCO 2021 meeting. The ongoing phase II trial included 17 patients, who were treated with 4–6 cycles of chemotherapy plus immunotherapy, followed by immunotherapy as maintenance. The preliminary results showed that the combination is feasible and effective, with 55.6% ORR and 88.9% disease control rate. The overall incidence of KN046-related treatment-emergent adverse events was 64.7%, with 29.4% grade 3 TRAE. The most common KN046-related treatment-emergent adverse events were alanine and aspartate aminotransferase increase (29.4% and 17.6%, respectively), nausea (17.6%), rash (17.6%), and diarrhea, pyrexia, vomiting or hyperphosphatasemia (11.8% each).

No immune checkpoint inhibitors or immunological agents are currently used or approved in the neo-adjuvant setting for PDAC in clinical practice. However, the results of the ongoing trials in this field are expected and are summarized in Table 5.

### 3.4. The Role of the Microbiome in the Neo-Adjuvant Treatment of PDAC

The microbiome refers to a collection of micro-organisms and their genome in an environment [94]. Disruption of the microbiome has been implicated as a contributory factor in many disease states, including cancer [95]. Microbial diversity can arise due to modifiable host factors such as diet, antibiotic use, exercise, and environmental stressors. Additionally, microbial diversity is also due to genetic and demographic factors and the influence of hormones and bile acids. The study of the microbiome and its role as a possible prognostic biomarker, along with the potential for its modulation to improve the efficacy and toxicity of existing treatments, has gained significant interest in oncology. In PDAC, it has been suggested that the relatively disappointing results seen with immunotherapy may in some part be attributable to the patient microbiome [96].

The study conducted by Riquelme et al. provides supporting evidence for the composition of the microbiome as a prognostic biomarker in PDAC as well as the role of modulating the tumor microbiome in improving clinical outcome [97]. They used a discovery and validation cohort and compared bacterial DNA from resected pancreatic tumor specimens in long-term survivors (median 10.1 years) and short-term survivors (<5 years from surgery). They ensured that the patients from the short- and long-term survivor groups were matched with regard to tumor stage, demographic characteristics, and the use of prior therapies, including neoadjuvant treatment and antibiotic use. Alpha diversity of the tumor microbiome was significantly higher in the long-term survivors than the short-term survivors in both the discovery and validation group. Moreover, an intra-tumoral microbiome signature was identified for long-term survivors. With this in mind, using animal models, fecal microbiota transplantation from humans to mice was conducted and influenced tumor growth and immune infiltration, thereby suggesting that modulation of the composition of the tumor microbiome may affect clinical outcomes. Whilst these data are preliminary, the encouraging results suggest that it is an area that requires further study.

Modulation of the microbiome is an exciting area of study, and other than fecal microbiota transplantation, antibiotics, prebiotics, and probiotics have been investigated, but results have been controversial, with survival benefit reported for macrolide antibiotic use in metastatic PDAC [98] but a detrimental impact on survival for tetracycline use in resected PDAC [99]. Specifically in the neo-adjuvant setting, neo-adjuvant chemotherapy has been shown to alter the biliary microbiome in PDAC. Whilst biliary stents may be associated with complications, in patients with a high bilirubin, biliary drainage needs to be established for neo-adjuvant chemotherapy to be feasible. Studies did not discuss the overall impact of the altered biliary microbiome from neo-adjuvant chemotherapy on survival [100,101]. However, they both recommend antibiotic prophylaxis in the peri-operative period, and although one study concludes that patients who did not receive neo-adjuvant chemotherapy were more likely to grow cephalosporin resistant pathogens [101], the other suggests that patients who had neo-adjuvant treatment were more likely to be resistant to cephalosporins [100]. In light of the conflicting results and limited studies available on this topic, the exact role of the biliary microbiome and the potential to modulate this for therapeutic benefit remains to be determined.

## 4. Discussion

Despite the recent improvements in surgical techniques and developments in the therapeutic armamentarium, PDAC still represents one of the most challenging tumors to treat in oncology. Surgical resection is generally feasible in very few patients at the time of diagnosis and remains the only chance of cure. However, resection alone is not sufficient, resulting in low cure rates with long-term survival generally not exceeding 20%. Therefore, other complementary treatments such as chemotherapy and radiotherapy need to be considered. It has been shown that either option can individually add benefit in the pre- or post-operative setting. The optimal integration of such different strategies urgently needs to be defined.

Based on different series, up to 50% of resected patients never receive adjuvant treatments. Hence, focusing on delivering all the best available treatments before surgery is becoming increasingly important. Even more crucial is defining the optimal multimodal approach, with a combination of all the different treatment strategies likely to offer the most benefit for patients. In this regard, the so-called “total neo-adjuvant” approach looks very promising. It allows upfront delivery of what is regarded as the best polychemotherapy regimen (i.e., FOLFIRINOX), followed by radio-chemotherapy and ultimately surgery. This strategy has been associated with impressively high R0 resection rates that translated into significant PFS and OS improvements. As of today, this unarguably represents the most effective treatment strategy, but several questions still remain unanswered.

Specifically, it will be interesting to evaluate the performance of newer potentially less toxic cytotoxic agents or their best combination and the possible association with immunotherapy (i.e., immune checkpoint inhibitors and vaccines) with both chemo- and radiotherapy. Additionally, the integration of biomarkers to select which patients may benefit from different treatment strategies may play an increasingly important role. For example, molecular signatures for platinum sensitivity may better define which chemotherapy regimen should be used in this setting. Modulation of the tumor microbiome for therapeutic gain is another area that warrants further research in this setting.

Another key research avenue should also explore the role of the different available radiotherapy techniques and modalities and the role of immune priming in enhancing response. In particular, it will be important to define the value of SBRT incorporation as an alternative approach to conventionally fractionated external beam radiotherapy with concurrent chemotherapy due to its capability to deliver higher doses while reducing toxicity to normal tissues. Similarly, the use of smaller treatment fields, along with more conformal techniques and hypofractionated protocols, will hopefully improve final outcomes.

## 5. Conclusions

Ultimately, well designed randomized clinical trials will define the best sequence and duration of different treatment modalities, the optimal patient selection for surgical resection, and the need for post-operative adjuvant chemotherapy. The incorporation of standardized resectability criteria across all studies, along with the integration of translational research, is likely to result in improved outcomes.

## Figures and Tables

**Table 1:** Resectability criteria for PDAC.

Guidelines	Resectable	Borderline Resectable	Locally Advanced
NCCN [10]	No tumor contact: CA, SMA, CHA, SMV, or PV, or ≤180° contact without vein contour irregularity	Contact with SMV or PV of >180° or contact ≤180° with thrombosis of the vein with suitable vessel proximal and distal to the affected site. Contact with IVC. Pancreatic head/uncinate process: tumor contact with CHA without extension to CA or HA bifurcation. Contact with SMA of ≤180°. Pancreatic body/tail: contact with the CA of ≤180°, contact > 180° without involvement of the aorta and with intact, uninvolved gastroduodenal artery.	SMV/PV that cannot be reconstructed due to tumor involvement or vessel occlusion due to thrombus or tumor. Pancreatic head/uncinate process: contact with SMA >180°, contact with the CA of > 180°. Pancreatic body/tail: contact >180 ° with SMA or CA, contact with the CA and aortic involvement.
IAP Consensus Definition [11]	No tumor contact SMV, PV, SMA, CA, CHA	Tumor contact ≥180° with SMV/PV or bilateral narrowing/occlusion, not exceeding the inferior border of the duodenum with no tumor contact with SMA, CA, or CHA. Tumor contact of <180° with SMA, CA but without showing deformity/stenosis. Tumor contact with CHA without tumor contact of the PHA and/or CA. Biological criteria: CA19-9 > 500 IU/mL, regional lymph node metastasis on biopsy or PET-CT. Conditional criterion: PS ≥ 2.	SMV/PV: bilateral narrowing/occlusion, beyond the inferior border of the duodenum. Tumor contact/invasion with SMA, CA of ≥180°. CHA: tumor contact/invasion showing tumor contact/invasion of the PHA and/or CA. Tumor contact or invasion of aorta.

Legend. NCCN: National Comprehensive Cancer Network; CA: celiac axis; SMA: superior mesenteric artery; CHA: common hepatic artery; SMV: superior mesenteric vein; PV: portal vein; HA: hepatic artery; IVC: inferior vena cava; IAP: International Association of Pancreatology; PHA: proper hepatic artery; PS: performance status; PET-CT: positron emission tomography–computerized tomography.

**Table 2:** Key Phase III Studies of Adjuvant Treatment in PDAC.

Study	Population	*N*	Comparators	Primary Endpoint	Other Outcomes
ESPAC-1 [22,23]	R0 or R1 resection	541	CRT: 20 Gy tumor dose in ten daily fractions over 2 weeks with 500 mg/m^2^ 5-FU IV bolus d 1–3, repeated after a planned break of 2 weeks. CT: IV bolus FA 20 mg/m^2^ followed by IV bolus 5-FU 425 mg/m^2^ d 1–5 every 28 d for six cycles.	mOS: 15.5 mo for CRT vs. 16.1 mo for no CRT, *p* = 0.24 mOS: 19.7 mo for CT vs. 14.0 for no CT, *p* = 0.0005	5-year survival: 10% CRT vs. 20% no CRT, *p* = 0.05 5-year survival: 21% CT vs. 8% no CT, *p* = 0.009
CONKO-001 [24,25]	KPS ≥ 50% R0 or R1 resection Post-operative CEA/CA19-9 > 2.5 times ULN were excluded	368	Gem: 1 g/m^2^ d 1, 8, and 15 IV every 4 weeks for 6 mo (A) vs. Obs: (B).	DFS: 13.4 mo (A) vs. 6.7 mo (B), *p* < 0.001	mOS: 22.8 mo (A) vs. 20.2 mo (B), *p* = 0.01 5- and 10-year survival: 20.7% and 12.2% (A) vs. 10.4% and 7.7% (B) 5- and 10-year DFS: 16.6% and 14.3% (A) vs. 7.0% and 5.8% (B)
ESPAC-3 [26]	WHO PS ≤ 2, R0 or R1 resection	1149	5-FU+FA: FA, 20 mg/m^2^, IV bolus, followed by 5-FU, 425 mg/m^2^ IV bolus d 1–5 every 28 d (A) vs. Gem: 1 g/m^2^ d 1, 8, and 15 IV every 4 weeks for 6 mo (B).	mOS: 23 mo (A) vs. 23.6 mo (B), *p* = 0.39	DFS: 14.1 mo (A) vs. 14.3 mo (B) *p* = 0.53 1- and 2-year survival: 78.5% and 48.1% (A) vs. 80.1% and 49.1% (B)
ESPAC-4 [27,28]	WHO PS ≤ 2, R0 or R1 resection	732	Gem: 1 g/m^2^ d 1, 8, and 15 IV every 4 weeks for 6 mo (A) vs. Gem+Cap: Gem with Cap orally for 21 days followed by 7 days’ rest for 6 mo at a daily dose of 1660 mg/m^2^.	mOS: 25.2 mo (A) vs. 28.0 mo (B), *p* = 0.032	RFS: 13.1 mo (A) vs. 13.9 (B), *p* = 0.082 3-year RFS: 20.9% (A) vs. 23.8% (B) 5-year RFS: 11.9% (A) vs. 18.6% (B) 5-year survival: 20.0% (A) vs. 28.0% (B), *p* = 0.049
PRODIGE-24 [18]	WHO PS ≤ 1 R0 or R1 resection Post-operative CA-19.9 ≤ 180 U/mL	493	Gem: 1 g/m^2^ d 1, 8, and 15 IV every 4 weeks for 6 mo (A) vs. mFOLFIRINOX: oxaliplatin 85 mg/m^2^, irinotecan 150 mg/m^2^, leucovorin 400 mg/m^2^, and 5-FU 2400 mg/m^2^ every 2 weeks (B).	DFS: 12.8 mo (A) vs. 21.6 mo (B), *p* < 0.001	mOS: 35.0 mo (A) vs. 54.4 mo (B), *p* = 0.003 3-year survival: 48.6% (A) vs. 63.4% (B) 1-, 2-, and 3-year DFS: 53.7%, 30.7%, 21.4% (A) vs. 69.0%, 47.0%, 39.7% (B) MFS: 17.7 mo (A) vs. 30.4 mo (B), *p* < 0.001
APACT [20,21]	WHO R0 or R1 resection Post-operative CA-19.9 < 100 U/mL	866	Gem: 1 g/m^2^ d 1, 8, and 15 IV every 4 weeks for 6 mo (A) vs. Gem/Nab-Paclitaxel: Gem with Nab-Paclitaxel 125 mg/m^2^ d 1, 8, and 15 IV every 4 weeks (B).	DFS: 18.8 mo (A) vs. 19.4 mo (B), *p* = 0.182	mOS: 37.7 mo (A) vs. 41.8 mo (B), *p* = 0.0091 5-year OS: 31% (A) vs. 38% (B)
JASPAC-01 [29]	WHO PS ≤ 1 R0 or R1 resection	385	Gem: 1 g/m^2^ d 1, 8, and 15 IV every 4 weeks for 6 mo (A) vs. S1: 40, 50, or 60 mg depending on BSA, orally for 28 d followed by a 14 d rest, every 6 weeks for up to 4 cycles.	mOS: 25.5 mo (A) vs. 46.5 mo (B), *p*_non-inferiority_ < 0.0001, *p* < 0.0001 for superiority	3- and 5-year OS: 38.8% and 24.4% (A) vs. 59.7 and 44.1% (B) RFS: 11.3 mo (A) vs. 22.9 mo, *p* < 0.0001

Legend. CRT: chemoradiotherapy; CT: chemotherapy; DFS: disease-free survival; mo: months; mOS: median overall survival; Obs: observation; WHO: World Health Organization; PS: performance status; KPS: Karnofsky performance status scale; Gem: gemcitabine; 5-FU: fluorouracil; FA: folinic acid; IV: intravenous; d: days; ULN: upper limit of normal; Cap: capecitaine; RFS: relapse-free survival; mFOLFIRINOX: modified FOLFIRINOX.

**Table 3:** Key Studies of Neo-adjuvant Chemoradiotherapy in PDAC.

Study Name (NCT Number)	Country	Phase	Population	*N*	Treatment	Primary Endpoint	Secondary Endpoints	Reference
PREOPANC-1 (NCT04927780)	The Netherlands	III R	Resectable/borderline resectable	246	Gemcitabine + RT → surgery → gemcitabine (A) vs. surgery → gemcitabine (B)	mOS 15.7 mo (A) vs. 14.3 mo (B), *p* = 0.025	Resection rate 61% (A) vs. 72% (B), *p* = 0.058 R0 resection rate 72% (A) vs. 43% (B), *p* < 0.001	[72,73]
FOLFIRINOX + RT for PDAC (NCT01591733)	USA	II	Borderline resectable	48	FOLFIRINOX → capecitabine + RT	R0 resection rate 65% (95% CI 49–78)	mPFS 14.7 mo mOS 37.7 mo	[74]
Alliance A021501 (NCT02839343)	USA	II R	Borderline resectable	126	FOLFIRINOX (A) vs. FOLFIRINOX → SBRT (B)	18-mo OS 67.9 (A) vs. 47.3% (B)	pts with pancreatectomy 18 mo OS 93.1 (A) vs. 78.9% (B) mOS 31.0 vs. 17.1 mo	[75]
ESPAC5F (CTI89500674)	UK	II R	Borderline resectable	90	Surgery → Gem/Cap (A) vs. Gem/Cap or FOLFIRINOX or CRT → surgery → Chemotherapy (B)	Resection rate 62% (A) vs. 55% (B), *p* = 0.668	R0 resection rate 15% (A) vs. 23% (B), *p* = 0.721 1-year survival: 42% (A) vs. 77% (B), *p* < 0.001	[76]

Legend. CI: confidence interval; mo: months; mOS: median overall survival; mPFS: median progression-free survival; OS: overall survival; pts: patients; R: randomized; R0: resection margins free; RR: response rate; RT: radiotherapy; SBRT: stereotactic body radiation therapy; y: year; Gem/Cap: gemcitabine/capecitabine.

**Table 4:** Studies of Neo-adjuvant Chemotherapy in Locally Advanced PDAC.

Study Name (NCT Number)	Country	Phase	Selected Population	*N*	Treatment	Primary Endpoint	Secondary Endpoints	Reference
GAP (NCT02043730)	Italy	II R	Locally advanced unresectable	124	Nab-paclitaxel + gemcitabine (A) vs. gemcitabine (B)	PDR3 25.4 (A) vs. 45.6% (B), *p* = 0.01 PDR6 20.8 (A) vs. 35.6% (B)	RR 27 (A) vs. 5.3% (B) mPFS 7 mo (A) vs. 4 mo (B) mOS 12.7 (A) vs. 10.6 mo (B)	[79]
LAPACT (NCT02301143)	Western	II	Locally advanced	107	Nab-paclitaxel + gemcitabine	mTTF 9.0 mo (90% CI 7.3–10.1)	mPFS 10.9 mo mOS 18.8 mo DCR 77.6% Resection in 17 (16%)	[80]
JCOG1407 (jRCTs031180085)	Japan	II R	Locally advanced	126	FOLFIRINOX (A) vs. nab-paclitaxel + gemcitabine (B)	1 y OS 77.4 (A) vs. 82.5% (B) 2 y OS 48.2 (A) vs. 39.7% (B)	mOS 2 y (A) vs. 1.8 y (B) PFS 1 y 47.5 (A) vs. 40.2% (B) MFS 1 y 64.2 (A) vs. 57.3% (B) RR 30.9 (A) vs. 41.4% (B)	[82]

Legend. CI: confidence interval; DCR: disease control rate; mo: months; MFS: metastases-free survival; mo: months; mOS: median overall survival; mPFS: median progression-free survival; mTTF: median time to treatment failure; N: number of patients; PDR3: reduction in progressive disease at three months; PDR6: reduction in progressive disease at six months; R: randomized; R0: resection margins free; RR: response rate; y: year.

**Table 5:** Ongoing studies with neo-adjuvant immunotherapy in resectable and locally advanced PDAC.

Study Name (NCT Number)	Country	Phase	Selected Population	*N*	Drugs	Primary Endpoint	Secondary Endpoints
NCT03983057	China	III R	Locally advanced/borderline resectable	830	mFOLFIRINOX vs. mFOLFIRINOX + anti-PD-1 Ab	PFS	RR R0 rate ORR DCR OS AEs CA 19.9 EORTC QLQ-PAN26 score
NCT03161379	USA	II	Locally advanced	30	GVAX vaccine + cyclophosphamide + nivolumab + SBRT	CD8 count in tumor microenvironment	pCR rate
NCT02305186	USA	Ib/II R	Resectable/borderline resectable	68	Pembrolizumab + capecitabine-RT vs. capecitabine-RT	TILs per HPF in resected tissue DLTs	DFS OS RR
NCT04327986	USA	I/II	Borderline resectable/locally advanced unresectable	126	Arm 1: M9241 (immunocytokine) + M7824 (ICI) Arm 2: M9241 + M7824-SBRT Arm 3: M7824 + M9241-SBRT	BOR	RP2D
NCT03970252	USA	I/II	Borderline resectable	36	mFOLFIRINOX + nivolumab	Clinically relevant pancreatic fistula in post-operative period	pCR rate
NCT03373188	USA	I R	Resectable stage I–III	32	Surgery vs. VX15/2503 (anti-SEMA4D) vs. VX15/2503 + ipilimumab vs. VX15/2503 + nivolumab	CD8 T cell infiltration	AEs

Legend. AEs: adverse events; BOR: best overall response; DCR: disease control rate; DFS: disease-free survival; DLT: dose-limiting toxicity; HPF: high-power field; ICI: immune checkpoint inhibitor; ORR: overall response rate; OS: overall survival; pCR: pathological complete response; R0: resection margins free; R: randomized; RP2D: recommended phase II dose; RR: response rate; RT: radiotherapy; SBRT: stereotactic body radiation therapy; TILs: tumor-infiltrating lymphocytes.

## References

[1] Sung H., Ferlay J., Siegel R.L., Laversanne M., Soerjomataram I., Jemal A., Bray F. (2021). Global Cancer Statistics 2020: GLOBOCAN Estimates of Incidence and Mortality Worldwide for 36 Cancers in 185 Countries. CA Cancer J. Clin..

[2] International Agency for Research on Cancer Cancer Today. https://gco.iarc.fr/today/online-analysis-map?v=2020&mode=population&mode_population=continents&population=900&populations=900&key=asr&sex=0&cancer=13&type=0&statistic=5&prevalence=0&population_group=0&ages_group%5B%5D=0&ages_group%5B%5D=17&nb_items=10&gr.

[3] Ferlay J., Colombet M., Soerjomataram I., Dyba T., Randi G., Bettio M., Gavin A., Visser O., Bray F. (2018). Cancer incidence and mortality patterns in Europe: Estimates for 40 countries and 25 major cancers in 2018. Eur. J. Cancer.

[4] Rahib L., Smith B.D., Aizenberg R., Rosenzweig A.B., Fleshman J.M., Matrisian L.M. (2014). Projecting cancer incidence and deaths to 2030: The unexpected burden of thyroid, liver, and pancreas cancers in the united states. Cancer Res..

[5] Carioli G., Malvezzi M., Bertuccio P., Boffetta P., Levi F., La Vecchia C., Negri E. (2021). European cancer mortality predictions for the year 2021 with focus on pancreatic and female lung cancer. Ann. Oncol..

[6] Sahin I.H., Iacobuzio-Donahue C.A., O’Reilly E.M. (2016). Molecular signature of pancreatic adenocarcinoma: An insight from genotype to phenotype and challenges for targeted therapy. Expert Opin. Ther. Targets.

[7] Ducreux M., Cuhna A.S., Caramella C., Hollebecque A., Burtin P., Goéré D., Seufferlein T., Haustermans K., Van Laethem J.L., Conroy T. (2015). Cancer of the pancreas: ESMO Clinical Practice Guidelines for diagnosis, treatment and follow-up. Ann. Oncol..

[8] Navez J., Bouchart C., Lorenzo D., Bali M.A., Closset J., van Laethem J.-L. (2021). What Should Guide the Performance of Venous Resection During Pancreaticoduodenectomy for Pancreatic Ductal Adenocarcinoma with Venous Contact?. Ann. Surg. Oncol..

[9] Ouaïssi M., Turrini O., Hubert C., Louis G., Gigot J.-F., Mabrut J.-Y. (2014). Vascular resection during radical resection of pancreatic adenocarcinomas: Evolution over the past 15 years. J. Hepatobil. Pancreat. Sci..

[10] (2020). NCCN Clinical Practice Guidelines in Oncology Pancreatic Adenocarcinoma Version 1. https://www2.tri-kobe.org/nccn/guideline/archive/pancreas2020/english/pancreatic.pdf.

[11] Isaji S., Mizuno S., Windsor J.A., Bassi C., Fernández-del Castillo C., Hackert T., Hayasaki A., Katz M.H.G., Kim S.-W., Kishiwada M. (2018). International consensus on definition and criteria of borderline resectable pancreatic ductal adenocarcinoma 2017. Pancreatology.

[12] Callery M.P., Chang K.J., Fishman E.K., Talamonti M.S., William Traverso L., Linehan D.C. (2009). Pretreatment Assessment of Resectable and Borderline Resectable Pancreatic Cancer: Expert Consensus Statement. Ann. Surg. Oncol..

[13] Katz M.H.G., Pisters P.W.T., Evans D.B., Sun C.C., Lee J.E., Fleming J.B., Vauthey J.N., Abdalla E.K., Crane C.H., Wolff R.A. (2008). Borderline Resectable Pancreatic Cancer: The Importance of This Emerging Stage of Disease. J. Am. Coll. Surg..

[14] Varadhachary G.R., Tamm E.P., Abbruzzese J.L., Xiong H.Q., Crane C.H., Wang H., Lee J.E., Pisters P.W.T., Evans D.B., Wolff R.A. (2006). Borderline Resectable Pancreatic Cancer: Definitions, Management, and Role of Preoperative Therapy. Ann. Surg. Oncol..

[15] Katz M.H.G., Marsh R., Herman J.M., Shi Q., Collison E., Venook A.P., Kindler H.L., Alberts S.R., Philip P., Lowy A.M. (2013). Borderline Resectable Pancreatic Cancer: Need for Standardization and Methods for Optimal Clinical Trial Design. Ann. Surg. Oncol..

[16] Bengtsson A., Andersson R., Ansari D. (2020). The actual 5-year survivors of pancreatic ductal adenocarcinoma based on real-world data. Sci. Rep..

[17] Sener S.F., Fremgen A., Menck H.R., Winchester D.P. (1999). Pancreatic cancer: A report of treatment and survival trends for 100,313 patients diagnosed from 1985–1995, using the National Cancer Database 11 No competing interests declared. J. Am. Coll. Surg..

[18] Conroy T., Hammel P., Hebbar M., Ben Abdelghani M., Wei A.C., Raoul J.-L., Choné L., Francois E., Artru P., Biagi J.J. (2018). FOLFIRINOX or Gemcitabine as Adjuvant Therapy for Pancreatic Cancer. N. Engl. J. Med..

[19] Von Hoff D.D., Ervin T., Arena F.P., Chiorean E.G., Infante J., Moore M., Seay T., Tjulandin S.A., Ma W.W., Saleh M.N. (2013). Increased Survival in Pancreatic Cancer with nab-Paclitaxel plus Gemcitabine. N. Engl. J. Med..

[20] Tempero M.A., Reni M., Riess H., Pelzer U., O’Reilly E.M., Winter J.M., Oh D.-Y., Li C.-P., Tortora G., Chang H.-M. (2019). APACT: Phase III, multicenter, international, open-label, randomized trial of adjuvant nab-paclitaxel plus gemcitabine ( nab-P/G) vs gemcitabine (G) for surgically resected pancreatic adenocarcinoma. J. Clin. Oncol..

[21] Tempero M., O’Reilly E., Van Cutsem E., Berlin J., Philip P., Goldstein D., Tabernero J., Borad M., Bachet J., Parner V. (2021). LBA-1 Phase 3 APACT trial of adjuvant nab-paclitaxel plus gemcitabine (nab-P + Gem) vs gemcitabine (Gem) alone in patients with resected pancreatic cancer (PC): Updated 5-year overall survival. Ann. Oncol..

[22] Neoptolemos J.P., Dunn J.A., Stocken D.D., Almond J., Link K., Beger H., Bassi C., Falconi M., Pederzoli P., Dervenis C. (2001). Adjuvant chemoradiotherapy and chemotherapy in resectable pancreatic cancer: A randomised controlled trial. Lancet.

[23] Neoptolemos J.P., Stocken D.D., Friess H., Bassi C., Dunn J.A., Hickey H., Beger H., Fernandez-Cruz L., Dervenis C., Lacaine F. (2004). A Randomized Trial of Chemoradiotherapy and Chemotherapy after Resection of Pancreatic Cancer. N. Engl. J. Med..

[24] Oettle H., Post S., Neuhaus P., Gellert K., Langrehr J., Ridwelski K., Schramm H., Fahlke J., Zuelke C., Burkart C. (2007). Adjuvant Chemotherapy with Gemcitabine vs. Observation in Patients Undergoing Curative-Intent Resection of Pancreatic Cancer. JAMA.

[25] Oettle H., Neuhaus P., Hochhaus A., Hartmann J.T., Gellert K., Ridwelski K., Niedergethmann M., Zülke C., Fahlke J., Arning M.B. (2013). Adjuvant Chemotherapy with Gemcitabine and Long-term Outcomes Among Patients With Resected Pancreatic Cancer. JAMA.

[26] Neoptolemos J.P., Stocken D.D., Bassi C., Ghaneh P., Cunningham D., Goldstein D., Padbury R., Moore M.J., Gallinger S., Mariette C. (2010). Adjuvant Chemotherapy with Fluorouracil Plus Folinic Acid vs. Gemcitabine Following Pancreatic Cancer Resection. JAMA.

[27] Neoptolemos J.P., Palmer D.H., Ghaneh P., Psarelli E.E., Valle J.W., Halloran C.M., Faluyi O., O’Reilly D.A., Cunningham D., Wadsley J. (2017). Comparison of adjuvant gemcitabine and capecitabine with gemcitabine monotherapy in patients with resected pancreatic cancer (ESPAC-4): A multicentre, open-label, randomised, phase 3 trial. Lancet.

[28] Neoptolemos J.P., Palmer D.H., Ghaneh P., Valle J.W., Cunningham D., Wadsley J., Meyer T., Anthoney A., Glimelius B., Falk S. (2020). ESPAC-4: A multicenter, international, open-label randomized controlled phase III trial of adjuvant combination chemotherapy of gemcitabine (GEM) and capecitabine (CAP) vs. monotherapy gemcitabine in patients with resected pancreatic ductal adenocarcin. J. Clin. Oncol..

[29] Uesaka K., Boku N., Fukutomi A., Okamura Y., Konishi M., Matsumoto I., Kaneoka Y., Shimizu Y., Nakamori S., Sakamoto H. (2016). Adjuvant chemotherapy of S-1 versus gemcitabine for resected pancreatic cancer: A phase 3, open-label, randomised, non-inferiority trial (JASPAC 01). Lancet.

[30] Altman A.M., Wirth K., Marmor S., Lou E., Chang K., Hui J.Y.C., Tuttle T.M., Jensen E.H., Denbo J.W. (2019). Completion of Adjuvant Chemotherapy After Upfront Surgical Resection for Pancreatic Cancer Is Uncommon Yet Associated With Improved Survival. Ann. Surg. Oncol..

[31] Bilimoria K.Y., Bentrem D.J., Ko C.Y., Tomlinson J.S., Stewart A.K., Winchester D.P., Talamonti M.S. (2007). Multimodality therapy for pancreatic cancer in the U.S. Cancer.

[32] Ma S.J., Oladeru O.T., Miccio J.A., Iovoli A.J., Hermann G.M., Singh A.K. (2019). Association of Timing of Adjuvant Therapy with Survival in Patients With Resected Stage I to II Pancreatic Cancer. JAMA Netw. Open.

[33] Valle J.W., Palmer D., Jackson R., Cox T., Neoptolemos J.P., Ghaneh P., Rawcliffe C.L., Bassi C., Stocken D.D., Cunningham D. (2014). Optimal Duration and Timing of Adjuvant Chemotherapy After Definitive Surgery for Ductal Adenocarcinoma of the Pancreas: Ongoing Lessons From the ESPAC-3 Study. J. Clin. Oncol..

[34] Jones R.P., Psarelli E.-E., Jackson R., Ghaneh P., Halloran C.M., Palmer D.H., Campbell F., Valle J.W., Faluyi O., O’Reilly D.A. (2019). Patterns of Recurrence After Resection of Pancreatic Ductal Adenocarcinoma. JAMA Surg..

[35] Hanna-Sawires R.G., Schiphuis J.H., Wuhrer M., Vasen H.F.A., van Leerdam M.E., Bonsing B.A., Mesker W.E., van der Burgt Y.E.M., Tollenaar R.A.E.M. (2021). Clinical Perspective on Proteomic and Glycomic Biomarkers for Diagnosis, Prognosis, and Prediction of Pancreatic Cancer. Int. J. Mol. Sci..

[36] Xing H., Wang J., Wang Y., Tong M., Hu H., Huang C., Li D. (2018). Diagnostic Value of CA 19-9 and Carcinoembryonic Antigen for Pancreatic Cancer: A Meta-Analysis. Gastroenterol. Res. Pract..

[37] Park J., Lee E., Park K.-J., Park H.-D., Kim J.-W., Woo H.I., Lee K.H., Lee K.-T., Lee J.K., Park J.-O. (2017). Large-scale clinical validation of biomarkers for pancreatic cancer using a mass spectrometry-based proteomics approach. Oncotarget.

[38] Kim J., Bamlet W.R., Oberg A.L., Chaffee K.G., Donahue G., Cao X.-J., Chari S., Garcia B.A., Petersen G.M., Zaret K.S. (2017). Detection of early pancreatic ductal adenocarcinoma with thrombospondin-2 and CA19-9 blood markers. Sci. Transl. Med..

[39] Liu X., Zheng W., Wang W., Shen H., Liu L., Lou W., Wang X., Yang P. (2017). A new panel of pancreatic cancer biomarkers discovered using a mass spectrometry-based pipeline. Br. J. Cancer.

[40] Staal B., Liu Y., Barnett D., Hsueh P., He Z., Gao C., Partyka K., Hurd M.W., Singhi A.D., Drake R.R. (2019). The sTRA Plasma Biomarker: Blinded Validation of Improved Accuracy Over CA19-9 in Pancreatic Cancer Diagnosis. Clin. Cancer Res..

[41] Sefrioui D., Blanchard F., Toure E., Basile P., Beaussire L., Dolfus C., Perdrix A., Paresy M., Antonietti M., Iwanicki-Caron I. (2017). Diagnostic value of CA19.9, circulating tumour DNA and circulating tumour cells in patients with solid pancreatic tumours. Br. J. Cancer.

[42] Lee B., Lipton L., Cohen J., Tie J., Javed A.A., Li L., Goldstein D., Burge M., Cooray P., Nagrial A. (2019). Circulating tumor DNA as a potential marker of adjuvant chemotherapy benefit following surgery for localized pancreatic cancer. Ann. Oncol..

[43] Wang Z.-Y., Ding X.-Q., Zhu H., Wang R.-X., Pan X.-R., Tong J.-H. (2019). KRAS Mutant Allele Fraction in Circulating Cell-Free DNA Correlates with Clinical Stage in Pancreatic Cancer Patients. Front. Oncol..

[44] Bernard V., Kim D.U., San Lucas F.A., Castillo J., Allenson K., Mulu F.C., Stephens B.M., Huang J., Semaan A., Guerrero P.A. (2019). Circulating Nucleic Acids Are Associated with Outcomes of Patients With Pancreatic Cancer. Gastroenterology.

[45] Buscail E., Maulat C., Muscari F., Chiche L., Cordelier P., Dabernat S., Alix-Panabières C., Buscail L. (2019). Liquid Biopsy Approach for Pancreatic Ductal Adenocarcinoma. Cancers.

[46] Heredia-Soto V., Rodríguez-Salas N., Feliu J. (2021). Liquid Biopsy in Pancreatic Cancer: Are We Ready to Apply It in the Clinical Practice?. Cancers.

[47] Abdallah R., Taly V., Zhao S., Pietrasz D., Bachet J.-B., Basile D., Mas L., Zaanan A., Laurent-Puig P., Taieb J. (2020). Plasma circulating tumor DNA in pancreatic adenocarcinoma for screening, diagnosis, prognosis, treatment and follow-up: A systematic review. Cancer Treat. Rev..

[48] Zhu Y., Zhang H., Chen N., Hao J., Jin H., Ma X. (2020). Diagnostic value of various liquid biopsy methods for pancreatic cancer: A systematic review and meta-analysis. Medicine.

[49] Liu H., Qiao S., Fan X., Gu Y., Zhang Y., Huang S. (2021). Role of exosomes in pancreatic cancer. Oncol. Lett..

[50] Mortoglou M., Tabin Z.K., Arisan E.D., Kocher H.M., Uysal-Onganer P. (2021). Non-coding RNAs in pancreatic ductal adenocarcinoma: New approaches for better diagnosis and therapy. Transl. Oncol..

[51] Ikuta S., Aihara T., Yamanaka N. (2019). Preoperative C-reactive protein to albumin ratio is a predictor of survival after pancreatic resection for pancreatic ductal adenocarcinoma. Asia. Pac. J. Clin. Oncol..

[52] Dimitrakopoulos C., Vrugt B., Flury R., Schraml P., Knippschild U., Wild P., Hoerstrup S., Henne-Bruns D., Wuerl P., Graf R. (2019). Identification and Validation of a Biomarker Signature in Patients with Resectable Pancreatic Cancer via Genome-Wide Screening for Functional Genetic Variants. JAMA Surg..

[53] Miyake K., Mori R., Homma Y., Matsuyama R., Okayama A., Murakami T., Hirano H., Endo I. (2017). MZB1 in borderline resectable pancreatic cancer resected after neoadjuvant chemoradiotherapy. J. Surg. Res..

[54] Gao C., Wisniewski L., Liu Y., Staal B., Beddows I., Plenker D., Aldakkak M., Hall J., Barnett D., Gouda M.K. (2021). Detection of Chemotherapy-resistant Pancreatic Cancer Using a Glycan Biomarker, sTRA. Clin. Cancer Res..

[55] Lee S.H., Cho H.J., Kang I., Choi S.H., Lee S., Lee J.-S. (2021). Integrative multi-omics profiling of resectable pancreatic cancer reveals clinically relevant molecular subtypes with precision strategies beyond the clinical staging system. Ann. Hepato-Biliary-Pancreat. Surg..

[56] Chan-Seng-Yue M., Kim J.C., Wilson G.W., Ng K., Figueroa E.F., O’Kane G.M., Connor A.A., Denroche R.E., Grant R.C., McLeod J. (2020). Transcription phenotypes of pancreatic cancer are driven by genomic events during tumor evolution. Nat. Genet..

[57] Janssen Q.P., O’Reilly E.M., van Eijck C.H.J., Groot Koerkamp B. (2020). Neoadjuvant Treatment in Patients with Resectable and Borderline Resectable Pancreatic Cancer. Front. Oncol..

[58] Janssen Q.P., Buettner S., Suker M., Beumer B.R., Addeo P., Bachellier P., Bahary N., Bekaii-Saab T., Bali M.A., Besselink M.G. (2019). Neoadjuvant FOLFIRINOX in Patients with Borderline Resectable Pancreatic Cancer: A Systematic Review and Patient-Level Meta-Analysis. J. Natl. Cancer Inst..

[59] Strobel O., Lorenz P., Hinz U., Gaida M., König A.-K., Hank T., Niesen W., Kaiser J., Al-Saeedi M., Bergmann F. (2020). Actual Five-year Survival After Upfront Resection for Pancreatic Ductal Adenocarcinoma. Ann. Surg..

[60] Hartwig W., Strobel O., Hinz U., Fritz S., Hackert T., Roth C., Büchler M.W., Werner J. (2013). CA19-9 in Potentially Resectable Pancreatic Cancer: Perspective to Adjust Surgical and Perioperative Therapy. Ann. Surg. Oncol..

[61] Bergquist J.R., Puig C.A., Shubert C.R., Groeschl R.T., Habermann E.B., Kendrick M.L., Nagorney D.M., Smoot R.L., Farnell M.B., Truty M.J. (2016). Carbohydrate Antigen 19-9 Elevation in Anatomically Resectable, Early Stage Pancreatic Cancer Is Independently Associated with Decreased Overall Survival and an Indication for Neoadjuvant Therapy: A National Cancer Database Study. J. Am. Coll. Surg..

[62] Park S., Jang J.K., Byun J.H., Kim J.H., Lee S.S., Kim H.J., Hong S.B., Park S.H. (2021). CT in the prediction of margin-negative resection in pancreatic cancer following neoadjuvant treatment: A systematic review and meta-analysis. Eur. Radiol..

[63] Ta R., O’Connor D.B., Sulistijo A., Chung B., Conlon K.C. (2019). The Role of Staging Laparoscopy in Resectable and Borderline Resectable Pancreatic Cancer: A Systematic Review and Meta-Analysis. Dig. Surg..

[64] Truty M.J., Kendrick M.L., Nagorney D.M., Smoot R.L., Cleary S.P., Graham R.P., Goenka A.H., Hallemeier C.L., Haddock M.G., Harmsen W.S. (2021). Factors Predicting Response, Perioperative Outcomes, and Survival Following Total Neoadjuvant Therapy for Borderline/Locally Advanced Pancreatic Cancer. Ann. Surg..

[65] Barreto S.G., Loveday B., Windsor J.A., Pandanaboyana S. (2019). Detecting tumour response and predicting resectability after neoadjuvant therapy for borderline resectable and locally advanced pancreatic cancer. ANZ J. Surg..

[66] Primrose P.J. (2020). NICE Guidelines: Pancreatic cancer in adults: Diagnosis and management. Pancreatology.

[67] Ghaneh P., Hanson R., Titman A., Lancaster G., Plumpton C., Lloyd-Williams H., Yeo S.T., Edwards R.T., Johnson C., Abu Hilal M. (2018). PET-PANC: Multicentre prospective diagnostic accuracy and health economic analysis study of the impact of combined modality 18fluorine-2-fluoro-2-deoxy-d-glucose positron emission tomography with computed tomography scanning in the diagnosis and managemen. Health Technol. Assess..

[68] Tamburrino D., Riviere D., Yaghoobi M., Davidson B.R., Gurusamy K.S. (2016). Diagnostic accuracy of different imaging modalities following computed tomography (CT) scanning for assessing the resectability with curative intent in pancreatic and periampullary cancer. Cochrane Database Syst. Rev..

[69] Stessin A.M., Meyer J.E., Sherr D.L. (2008). Neoadjuvant Radiation is Associated with Improved Survival in Patients With Resectable Pancreatic Cancer: An Analysis of Data From the Surveillance, Epidemiology, and End Results (SEER) Registry. Int. J. Radiat. Oncol..

[70] Cloyd J.M., Chen H.-C., Wang X., Tzeng C.-W.D., Kim M.P., Aloia T.A., Vauthey J.-N., Lee J.E., Katz M.H.G. (2019). Chemotherapy vs. Chemoradiation as Preoperative Therapy for Resectable Pancreatic Ductal Adenocarcinoma. Pancreas.

[71] Versteijne E., Vogel J.A., Besselink M.G., Busch O.R.C., Wilmink J.W., Daams J.G., van Eijck C.H.J., Groot Koerkamp B., Rasch C.R.N., van Tienhoven G. (2018). Meta-analysis comparing upfront surgery with neoadjuvant treatment in patients with resectable or borderline resectable pancreatic cancer. Br. J. Surg..

[72] Van Eijck C.H.J., Versteijne E., Suker M., Groothuis K., Besselink M.G.H., Busch O.R.C., Bonsing B.A., Groot Koerkamp B., de Hingh I.H.J.T., Festen S. (2021). Preoperative chemoradiotherapy to improve overall survival in pancreatic cancer: Long-term results of the multicenter randomized phase III PREOPANC trial. J. Clin. Oncol..

[73] Versteijne E., Suker M., Groothuis K., Akkermans-Vogelaar J.M., Besselink M.G., Bonsing B.A., Buijsen J., Busch O.R., Creemers G.-J.M., van Dam R.M. (2020). Preoperative Chemoradiotherapy vs. Immediate Surgery for Resectable and Borderline Resectable Pancreatic Cancer: Results of the Dutch Randomized Phase III PREOPANC Trial. J. Clin. Oncol..

[74] Murphy J.E., Wo J.Y., Ryan D.P., Jiang W., Yeap B.Y., Drapek L.C., Blaszkowsky L.S., Kwak E.L., Allen J.N., Clark J.W. (2018). Total Neoadjuvant Therapy With FOLFIRINOX Followed by Individualized Chemoradiotherapy for Borderline Resectable Pancreatic Adenocarcinoma. JAMA Oncol..

[75] Katz M.H.G., Shi Q., Meyers J.P., Herman J.M., Choung M., Wolpin B.M., Ahmad S., Marsh R.d.W., Schwartz L.H., Behr S. (2021). Alliance A021501: Preoperative mFOLFIRINOX or mFOLFIRINOX plus hypofractionated radiation therapy (RT) for borderline resectable (BR) adenocarcinoma of the pancreas. J. Clin. Oncol..

[76] Ghaneh P., Palmer D.H., Cicconi S., Halloran C., Psarelli E.E., Rawcliffe C.L., Sripadam R., Mukherjee S., Wadsley J., Al-Mukhtar A. (2020). ESPAC-5F: Four-arm, prospective, multicenter, international randomized phase II trial of immediate surgery compared with neoadjuvant gemcitabine plus capecitabine (GEMCAP) or FOLFIRINOX or chemoradiotherapy (CRT) in patients with borderline resectable pan. J. Clin. Oncol..

[77] Conroy T., Desseigne F., Ychou M., Bouché O., Guimbaud R., Bécouarn Y., Adenis A., Raoul J.-L., Gourgou-Bourgade S., de la Fouchardière C. (2011). FOLFIRINOX versus Gemcitabine for Metastatic Pancreatic Cancer. N. Engl. J. Med..

[78] Suker M., Beumer B.R., Sadot E., Marthey L., Faris J.E., Mellon E.A., El-Rayes B.F., Wang-Gillam A., Lacy J., Hosein P.J. (2016). FOLFIRINOX for locally advanced pancreatic cancer: A systematic review and patient-level meta-analysis. Lancet Oncol..

[79] Cascinu S., Berardi R., Bianco R., Bilancia D., Zaniboni A., Ferrari D., Mosconi S., Spallanzani A., Cavanna L., Leo S. (2021). Nab-paclitaxel/gemcitabine combination is more effective than gemcitabine alone in locally advanced, unresectable pancreatic cancer—A GISCAD phase II randomized trial. Eur. J. Cancer.

[80] Philip P.A., Lacy J., Portales F., Sobrero A., Pazo-Cid R., Manzano Mozo J.L., Kim E.J., Dowden S., Zakari A., Borg C. (2020). Nab-paclitaxel plus gemcitabine in patients with locally advanced pancreatic cancer (LAPACT): A multicentre, open-label phase 2 study. Lancet Gastroenterol. Hepatol..

[81] Williet N., Petrillo A., Roth G., Ghidini M., Petrova M., Forestier J., Lopez A., Thoor A., Weislinger L., De Vita F. (2021). Gemcitabine/Nab-Paclitaxel versus FOLFIRINOX in Locally Advanced Pancreatic Cancer: A European Multicenter Study. Cancers.

[82] Ozaka M., Ueno M., Ishii H., Mizusawa J., Katayama H., Kataoka T., Okusaka T., Ikeda M., Miwa H., Kaneko S. (2021). Randomized phase II study of modified FOLFIRINOX vs. gemcitabine plus nab-paclitaxel combination therapy for locally advanced pancreatic cancer (JCOG1407). J. Clin. Oncol..

[83] Reni M., Balzano G., Zanon S., Zerbi A., Rimassa L., Castoldi R., Pinelli D., Mosconi S., Doglioni C., Chiaravalli M. (2018). Safety and efficacy of preoperative or postoperative chemotherapy for resectable pancreatic adenocarcinoma (PACT-15): A randomised, open-label, phase 2–3 trial. Lancet Gastroenterol. Hepatol..

[84] Ahmad S.A., Duong M., Sohal D.P.S., Gandhi N.S., Beg M.S., Wang-Gillam A., Wade J.L., Chiorean E.G., Guthrie K.A., Lowy A.M. (2020). Surgical Outcome Results From SWOG S1505. Ann. Surg..

[85] Sohal D., McDonough S., Ahmad S.A., Gandhi N., Beg M.S., Wang-Gillam A., Wade J.L., Guthrie K.A., Lowy A.M., Philip P.A. (2019). SWOG S1505: Initial findings on eligibility and neoadjuvant chemotherapy experience with mfolfirinox vs. gemcitabine/nab-paclitaxel for resectable pancreatic adenocarcinoma. J. Clin. Oncol..

[86] O’Kane G.M., Grünwald B.T., Jang G.-H., Masoomian M., Picardo S., Grant R.C., Denroche R.E., Zhang A., Wang Y., Lam B. (2020). GATA6 Expression Distinguishes Classical and Basal-like Subtypes in Advanced Pancreatic Cancer. Clin. Cancer Res..

[87] A Phase 0, Pre-Operative, Window-of-Opportunity Study to Assess Gene Expression in Patients with Resectable, Locally Advanced, or Metastatic Pancreatic Cancer (NEOPANC-01). https://pancreaticcancercanada.ca/press-release-neopancone-clinical-trial-launch/.

[88] Klein A.P. (2021). Pancreatic cancer epidemiology: Understanding the role of lifestyle and inherited risk factors. Nat. Rev. Gastroenterol. Hepatol..

[89] Golan T., Barenboim A., Lahat G., Nachmany I., Goykhman Y., Shacham-Shmueli E., Halpern N., Brazowski E., Geva R., Wolf I. (2020). Increased Rate of Complete Pathologic Response After Neoadjuvant FOLFIRINOX for BRCA Mutation Carriers with Borderline Resectable Pancreatic Cancer. Ann. Surg. Oncol..

[90] Ramnaraign B.H., Hughes S.J., Hitchcock K., Lee J.-H., Rogers S.C., Fan Z.H., Allegra C.J., Trevino J.G., El-Far A., Russell K.B. (2021). A phase II, open-label pilot study evaluating the safety and activity of liposomal irinotecan (Nal-IRI) in combination with 5-FU and oxaliplatin (NALIRIFOX) in preoperative treatment of pancreatic adenocarcinoma (NEO-Nal-IRI Study). J. Clin. Oncol..

[91] Wang-Gillam A., Li C.-P., Bodoky G., Dean A., Shan Y.-S., Jameson G., Macarulla T., Lee K.-H., Cunningham D., Blanc J.F. (2016). Nanoliposomal irinotecan with fluorouracil and folinic acid in metastatic pancreatic cancer after previous gemcitabine-based therapy (NAPOLI-1): A global, randomised, open-label, phase 3 trial. Lancet.

[92] Pompella L., Tirino G., Pappalardo A., Caterino M., Ventriglia A., Nacca V., Orditura M., Ciardiello F., De Vita F. (2020). Pancreatic Cancer Molecular Classifications: From Bulk Genomics to Single Cell Analysis. Int. J. Mol. Sci..

[93] Hewitt D.B., Nissen N., Hatoum H., Musher B., Seng J., Coveler A.L., Al-Rajabi R., Yeo C.J., Leiby B., Banks J. (2020). A Phase 3 Randomized Clinical Trial of Chemotherapy With or Without Algenpantucel-L (HyperAcute-Pancreas) Immunotherapy in Subjects with Borderline Resectable or Locally Advanced Unresectable Pancreatic Cancer. Ann. Surg..

[94] Berg G., Rybakova D., Fischer D., Cernava T., Vergès M.-C.C., Charles T., Chen X., Cocolin L., Eversole K., Corral G.H. (2020). Microbiome definition revisited: Old concepts and new challenges. Microbiome.

[95] Goodman B., Gardner H. (2018). The microbiome and cancer. J. Pathol..

[96] Abdul Rahman R., Lamarca A., Hubner R.A., Valle J.W., McNamara M.G. (2021). The Microbiome as a Potential Target for Therapeutic Manipulation in Pancreatic Cancer. Cancers.

[97] Riquelme E., Zhang Y., Zhang L., Montiel M., Zoltan M., Dong W., Quesada P., Sahin I., Chandra V., San Lucas A. (2019). Tumor Microbiome Diversity and Composition Influence Pancreatic Cancer Outcomes. Cell.

[98] Mohindroo C., Rogers J.E., Hasanov M., Mizrahi J., Overman M.J., Varadhachary G.R., Wolff R.A., Javle M.M., Fogelman D.R., Pant S. (2019). A retrospective analysis of antibiotics usage and effect on overall survival and progressive free survival in patients with metastatic pancreatic cancer. J. Clin. Oncol..

[99] Hasanov M., Mohindroo C., Rogers J., Prakash L., Overman M.J., Varadhachary G.R., Wolff R.A., Javle M.M., Fogelman D.R., Pant S. (2019). The effect of antibiotic use on survival of patients with resected pancreatic ductal adenocarcinoma. J. Clin. Oncol..

[100] Goel N., Nadler A., Reddy S., Hoffman J.P., Pitt H.A. (2019). Biliary microbiome in pancreatic cancer: Alterations with neoadjuvant therapy. HPB.

[101] Nadeem S.O., Jajja M.R., Maxwell D.W., Pouch S.M., Sarmiento J.M. (2021). Neoadjuvant chemotherapy for pancreatic cancer and changes in the biliary microbiome. Am. J. Surg..

